# Ant Colony-Based Hyperparameter Optimisation in Total Variation Reconstruction in X-ray Computed Tomography

**DOI:** 10.3390/s21020591

**Published:** 2021-01-15

**Authors:** Manasavee Lohvithee, Wenjuan Sun, Stephane Chretien, Manuchehr Soleimani

**Affiliations:** 1 Department of Nuclear Engineering, Faculty of Engineering, Chulalongkorn University, Bangkok 10330, Thailand; manasavee.lohvithee@bath.edu.uk; 2 National Physical Laboratory (NPL), Teddington, Middlesex TW11 0LW, UK; wenjuan.sun@npl.co.uk (W.S.); stephane.chretien@npl.co.uk (S.C.); 3 Laboratoire ERIC, Université Lyon 2, 69500 Bron, France; 4 Engineering Tomography Laboratory (ETL), University of Bath, Bath BA2 7AY, UK

**Keywords:** hyperparameter tuning, total variation (TV) regularization, iterative reconstruction, ant colony optimization, limited data X-ray CT, computer-aided hyperparameter selection, X-ray computed tomography, image reconstruction

## Abstract

In this paper, a computer-aided training method for hyperparameter selection of limited data X-ray computed tomography (XCT) reconstruction was proposed. The proposed method employed the ant colony optimisation (ACO) approach to assist in hyperparameter selection for the adaptive-weighted projection-controlled steepest descent (AwPCSD) algorithm, which is a total-variation (TV) based regularisation algorithm. During the implementation, there was a colony of artificial ants that swarm through the AwPCSD algorithm. Each ant chose a set of hyperparameters required for its iterative CT reconstruction and the correlation coefficient (CC) score was given for reconstructed images compared to the reference image. A colony of ants in one generation left a pheromone through its chosen path representing a choice of hyperparameters. Higher score means stronger pheromones/probabilities to attract more ants in the next generations. At the end of the implementation, the hyperparameter configuration with the highest score was chosen as an optimal set of hyperparameters. In the experimental results section, the reconstruction using hyperparameters from the proposed method was compared with results from three other cases: the conjugate gradient least square (CGLS), the AwPCSD algorithm using the set of arbitrary hyperparameters and the cross-validation method.The experiments showed that the results from the proposed method were superior to those of the CGLS algorithm and the AwPCSD algorithm using the set of arbitrary hyperparameters. Although the results of the ACO algorithm were slightly inferior to those of the cross-validation method as measured by the quantitative metrics, the ACO algorithm was over 10 times faster than cross—Validation. The optimal set of hyperparameters from the proposed method was also robust against an increase of noise in the data and can be applicable to different imaging samples with similar context. The ACO approach in the proposed method was able to identify optimal values of hyperparameters for a dataset and, as a result, produced a good quality reconstructed image from limited number of projection data. The proposed method in this work successfully solves a problem of hyperparameters selection, which is a major challenge in an implementation of TV based reconstruction algorithms.

## 1. Introduction

Despite the many advantages of using X-ray computed tomography (XCT) as a tool for medical analysis, the high radiation dose delivered to the patient during clinical exams remains a major concern. Reducing the radiation dose of XCT imaging has become a significant research topic. This can be implemented in two methods: one is to lower the photon flux in the XCT data acquisition process [1], the other is to reconstruct an XCT image from limited projection data [2,3]. The first method results in a high level of noise in the sinogram, whereas results from the second method suffer from artefacts when using conventional analytical reconstruction methods such as the filtered back-projection (FBP) algorithm [4]. Even though iterative methods are proven in many studies to produce good quality images when the projection data is not theoretically sufficient for exact image reconstruction [5,6], their implementations are mathematically more complex and computationally costly than analytical methods. When it comes to the success of limited data XCT, the total variation (TV) image reconstruction methods are shown to be superior in terms of handling the missing data. Efficient implementation of TV algorithm and their hyperparameter selection are critical for these algorithms to provide all the benefits to limited data CT. Missing data could happen for many reasons, but in medical application if we can produce the same images with fewer projections this could lead to lower dose to the patient. 

In our previous study [7], the adaptive-weighted projection-controlled steepest descent (AwPCSD) algorithm was proposed, which implements the edge-preserving function for cone-beam XCT (CBCT) reconstruction with limited data. This algorithm is able to address the problem of over-smoothing in the reconstructed image when using conventional TV norm as a regularization term. It also requires fewer hyperparameters for the implementation than the adaptive-steepest-descent projection onto convex sets (ASD-POCS) [8] approach, which significantly reduces the hyperparameter space. Apart from that, the work also pointed out how sensitive each hyperparameter is, and which ones affect the quality of the reconstructed image more than others. Some suggestions on the appropriate values for some hyperparameters were also given. The tuning of hyperparameters for TV-based XCT reconstruction is tedious work that requires an expert/experienced user to know which hyperparameter is needed to be adjusted in which direction. In addition, most of the hyperparameters are data-specific which makes it almost impossible to apply the same set of hyperparameters to data with different scanning scenarios, let alone different anatomical sites. Manual adjusting of the hyperparameters for iterative algorithms is commonly found in many studies [9,10]; a Freund and Shapire’s hedge approach was shown in [11].

To alleviate the difficulty of hyperparameter tuning for TV-based reconstruction algorithms, it is desirable to have an automatic algorithm that can identify an optimal set of hyperparameters. This has been an active area of research over the years and there have been many studies which have attempted to develop an automated hyperparameter tuning using different methods. Recently, Shen et al. [12] considered the case of iterative XCT reconstruction with TV minimization where each entry of TV hyperparameter controls the weight of an image pixel. They proposed a hyperparameter tuning policy network (PTPN) that employs deep reinforcement learning to train a system that can intelligently determine the direction and magnitude of each TV hyperparameter by observing an input image patch. In their study, the experiment was performed on fan-beam CT scanning geometry with 180 projections equally spaced over a 2π angular range.

The ant colony optimisation (ACO) approach is a probabilistic technique, which is used in computer science to solve computational problems such as finding good paths in discrete graphs [13]. Its use in hyperparameter optimisation in XCT reconstruction context is not extensive. Zheng et al. [14] employed this approach to learn the best hyperparameter setting in their iterative algorithm, which consisted of the ordered subset simultaneous iterative reconstruction technique (OS-SIRT) algorithm to constrain a data fidelity and a 2D filter with an unsharp masking for regularisation.

The contributions of this work can be explained as follows:

- The ACO approach for the complex non-linear problem of TV hyperparameter selection was used to select the optimal hyperparameters for the TV-regularised reconstruction algorithm. This work uses the AwPCSD algorithm [7] as a TV regularized reconstruction method, but any other TV regularizing algorithm can be tuned using the exact same approach. The reason for choosing this algorithm is that the AwPCSD algorithm implements the adaptive-weighted TV regularisation, which is able to preserve the edges of the reconstructed image better than alternatives with less sensitive hyperparameters required [7].
- The ACO approach in the proposed algorithm was able to identify optimal hyperparameters appropriate for a dataset and produce a good quality reconstructed image using limited numbers of projection data. The whole process does not require any knowledge about the iterative reconstruction algorithm from the user, nor the intervention during its implementation.
- The reconstructed image from the proposed algorithm was compared with the results from other ways of choosing hyperparameters and the conjugate gradient least square (CGLS) algorithm [15], visually and quantitatively. In addition, the optimal set of hyperparameters from the proposed algorithm was used to reconstruct images from the projection data with different levels of noise and different angle arrangements. The same set of experiments was also tested with different imaging samples to demonstrate the robustness of the proposed algorithm.

The main contribution of the paper is to develop a robust computer-aided and automatic parameter optimization in a TV-based XCT algorithm. The organization of this paper is as follows: in Section 2, the method of adaptive weighted TV minimization approach used to reconstruct image in this study is explained, as well as the hyperparameters required to implement the AwPCSD algorithm. The concept of the ACO approach to select the hyperparameters is also introduced in this section. In the first part of Section 3, the results from the training dataset of the 4D Extended Cardiac-Torso (XCAT) phantom [16,17] are presented. Then, the optimal setting of hyperparameters obtained from the training stage is tested with different samples of the XCAT phantom that have been parametrised differently. The results from the testing data are presented in the second part of Section 3 to show the robustness of the proposed algorithm. In Section 4, the conclusion is presented.

## 2. Background and Related Works

Over the past few years, a number of regularization terms have been introduced for under-sampled or noisy measurements such as TV, tight frame (TF) and nonlocal means (NLM) [12]. Minimization of TV norm as a regularization term is a common approach in many studies such as [2,8,18,19]. In their studies, a constrained TV minimization algorithm for image reconstruction in cone-beam CT is proposed. However, the main disadvantage of using TV as a regularization term is the over-smoothing of the reconstructed image which leads to the loss of low-contrast information [18,19]. Another concern of the TV-based regularization algorithms is that they are very sensitive to the hyperparameters, which are used to control the weights of the objective functions in the TV optimization problems. It is of the utmost importance to get these hyperparameters right in order to achieve the desired quality of image output.

### 2.1. The Total Variation Reconstruction Method

A typical minimization problem of iterative XCT reconstruction can be proposed as:

*x*^∗^ = *argmin_x_*||*Ax* − *b*||^2^ + *G*(*x*) (1)

where *x*^∗^ is an approximated solution, *x* is a vector representing a 3D image voxel in lexicographical order, *A* is a system matrix describing the intersections between each X-ray and the image voxels. Vector b represents the projection data measured on the image detectors at various projections. The first term of Equation (1) is the data consistency constraint which minimizes the discrepancy between a forward projection of the image to be reconstructed and measured projection data. The second term, *G*(*x*), is a regularization term, which reflects a priori information of the desired image. The regularization is added to reduce the space of possible solutions. As a regularization term in this study, we are interested in the minimization of the adaptive weighted TV (AwTV) norm of the image, as proposed by Liu et al. [19]. When the local voxel intensity difference is small, a strong weight can be given to emphasize the TV minimization of the non-edge region. Conversely, a weaker weight may be given for a larger voxel intensity difference to preserve the edge region of the image to be reconstructed.

The TV algorithm used is the AwPCSD algorithm that was developed earlier [7,8,11]. This algorithm is available in an open access software TIGRE toolbox: a MATLAB/Python GPU toolbox for X-ray CT image reconstruction [20]. The AwPCSD algorithm was developed to reconstruct the volumetric image from cone-beam CT projection data. The algorithm required two phases implemented alternately until the stopping criterion is satisfied.

The first phase is the iteration of the simultaneous algebraic reconstruction technique (SART) [21] to enforce the data-consistency according to the following two constraints:

I. data fitting condition
  |*Ax* − *b*| ≤ *ε* (2)
  where *ε* is an error bound that defines the amount of acceptable error between predicted and observed projection data.
II. non-negativity constraint
  *x* ≥ 0 (3)

The second phase is TV optimization, which is performed by the adaptive steepest descent to minimize the AwTV norm of the image. The step size in TV optimization phase is automatically computed in the AwPCSD algorithm. The AwPCSD algorithm is able to preserve the edges of the reconstructed image with small numbers of hyperparameters to calibrate. The following pseudo-code in Algorithm 1 summarizes the structure of the AwPCSD algorithm.
**Algorithm 1:** Pseudo code for the Adaptive-weighted Projection-Controlled Steepest Descent (AwPCSD) algorithm**Inputs:***x*^0^, *β*, *β*_red_, *ε*, *ng*, *δ*; **Procedure:** Set *w* = 0, *𝜂* = 1, *k* = 1; **while** stopping criteria not met      **for**
*w* = 1: *w_t_*
**do**      $x_{SART}=\;x^{\left(w\right)};$      $\nabla_{p}^{\left(w\right)}=\left|\left|Ax_{SART}-b\right|\right|;$      **if**
$(\nabla_{p}^{\left(w\right)}{}^{2}>$
*ε*) **then**      **for**
*n_angles_*
**do**     $x_{SART}=\;x_{SART}+\beta V^{-1}A^{T}W\left(\vec{b}-A\vec{x}\right);$
     **end for** **end if**             $x_{POCS}=\max\left(0,x_{SART}\right);$
    $\beta =\beta \times \;\beta_{\mathrm{red}}$
;    *n* = 0;             $x_{AwTV}^{\left(0\right)}=x_{POCS};$
**if** ($w>0\;\mathrm{and}\;\nabla_{p}^{\left(1\right)}{}^{2}>\varepsilon$) **then**    $\eta =k\left(\frac{\nabla_{p}^{\left(w\right)}}{\nabla_{p}^{\left(I\right)}}\right);$ **end if**     $s^{\left(n\right)}=\left(\frac{\partial {\left|\left|x^{\left(n\right)}\right|\right|}_{AwTV}}{\partial x^{\left(n\right)}}\right);$     **for** number of sub-iteration = 1:ng **do**      $x_{\mathrm{AwTV}}^{n+1}=x_{\mathrm{AwTV}}^{n}-\eta \times \frac{s^{\left(n\right)}}{\left|\left|s^{\left(n\right)}\right|\right|};$    **end for** **end for** $x^{\left(w\right)}=x_{\mathrm{AwTV}}^{n_{t}}\;$; Until stopping criteria are met **end while** **Output:**
$x^{\left(w\right)}$


### 2.2. Hyperparameters for Total Variation-Based Reconstruction Algorithms

The AwPCSD algorithm [7,11] requires the following five hyperparameters to implement the algorithm:

#### 2.2.1. Data-Inconsistency-Tolerance Parameter (*ε*)

The *ε* hyperparameter specifies the maximum *L*_2_ error to accept image as valid. As expressed in Equation (2), the value of this hyperparameter controls the level of data-consistency between the predicted and observed projection data. This hyperparameter is used as one of the stopping criteria of the TV algorithm. The algorithm ceases its implementation when the currently estimated image satisfies the following condition:

*c* < −0.99 and *dd* ≤ *ε* (4)

where *c* is the cosine of the angle between the TV and data-constraint gradients, *dd* is the *L*_2_ error between the measured projections and the projections computed from the estimated image in the current iteration.

#### 2.2.2. Total Variation Sub-Iteration Number (ng)

The *ng* hyperparameter specifies a number of sub-iteration that the TV optimisation phase is performed in each iteration of the algorithm

#### 2.2.3. Relaxation Parameter (*β*)

This is the hyperparameter that the SART process depends on. The value of *β* starts from 1.0 and slowly decreases until it reaches 0.0 according to the value of the next hyperparameter, *β*_red_.

#### 2.2.4. Reduction Factor of Relaxation Parameter (*β*_red_)

This hyperparameter reduces the value of *β* in the SART phase as the algorithm proceeds to the next iteration. The recommended setting for *β*_red_ found in our previous work is a value larger than 0.98 but smaller than 1. The *β* and *β*_red_ hyperparameters involve in another stopping criterion, following this condition:

β < 0.005 (5)

The algorithm stops when the value of *β* falls below 0.005 as no further difference between the reconstructed images of the current and adjacent iteration can be noticed.

#### 2.2.5. Scale Factor for Adaptive-Weighted Total Variation Norm (*δ*)

The *δ* hyperparameter controls the strength of the diffusion in the AwTV norm during each iteration [22]. The AwTV norm makes it possible to consider the gradient of the desired image and to take into account the changes of local voxel intensities.

## 3. Proposed Approach: Hyperparameter Tuning Method Using Ant Colony Optimisation (ACO)

The computer-aided hyperparameter tuning method for TV-based XCT reconstruction algorithm for limited data is proposed in this work. As explained in the previous section, the five hyperparameters required for the AwPCSD algorithm need to be selected properly for the reconstruction to work at its best. Some hyperparameters are set based on findings from our previous work [7], i.e., relaxation parameter (*β*) is set to 1, reduction factor of relaxation parameter (*β*_red_) is set to 0.99 and scale factor for adaptive-weighted TV norm (*δ*) is set to the 90th percentile of the histogram of an image reconstructed using the OS-SART algorithm. Apart from these, there are still two hyperparameters, the data-inconsistency-tolerance parameter (*ε*) and the TV sub iteration number (*ng*) that require proper setting.

To identify the proper values of *ε* and ng, we employ the ACO algorithm [13] to select the optimal set of hyperparameters for a given set of limited projection data. The ACO is a swarm intelligence approach to search for good paths in discrete graphs, as proposed in [13]. It is a modification of the Ant System algorithm, which was first introduced by Dorigo et al. in [23,24]. The ACO approach is a probabilistic technique and has been successfully implemented in numerous optimisation problems as reported in [13]. It belongs to the class of metaheuristic approaches, which can provide approximate solutions when the global optimum is unobtainable due to incomplete information. Its use in hyperparameter optimisation in the CT reconstruction context is not extensive. As briefly mentioned earlier, Zheng et al. [14] employed this approach to learn the best hyperparameter setting in their iterative algorithm.

**Figure 1:**
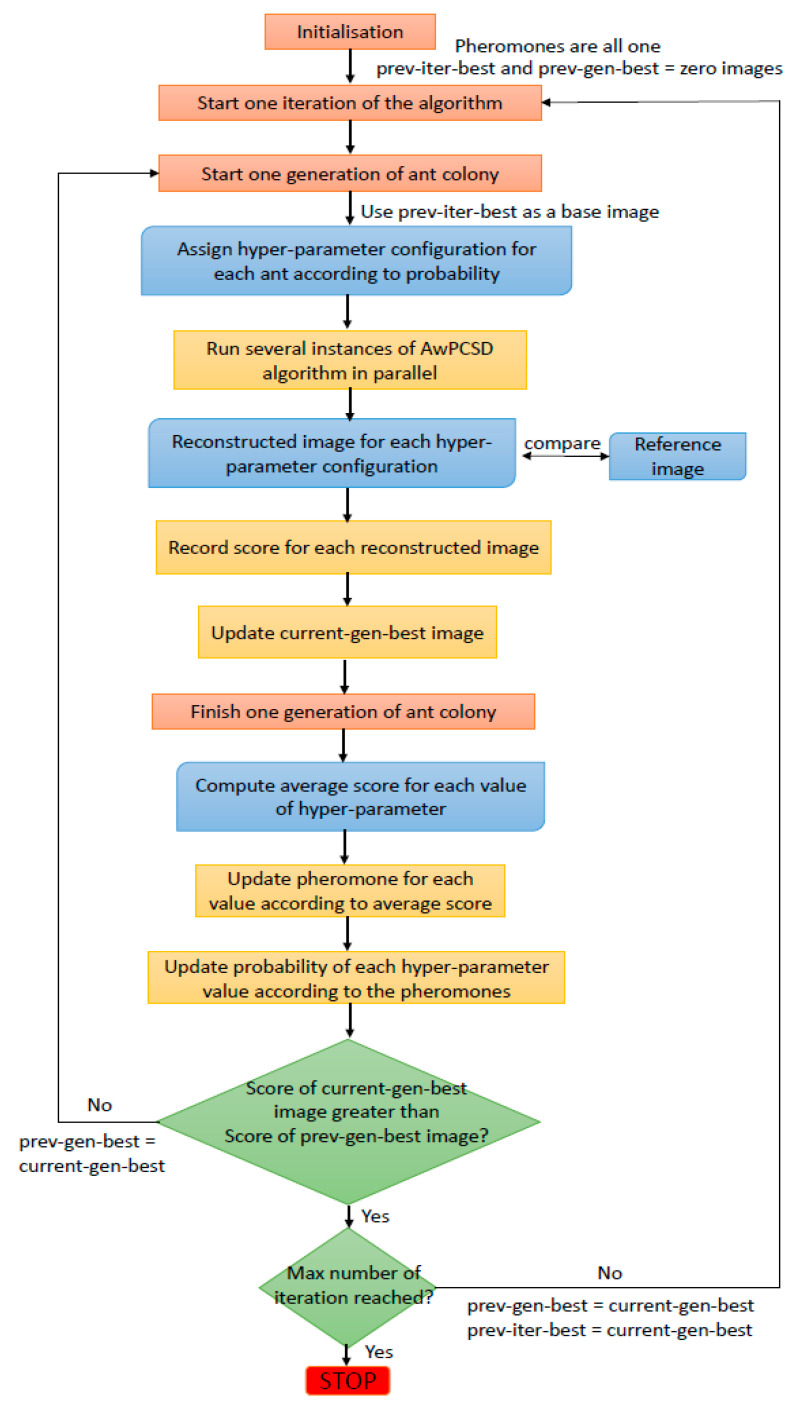
The framework of the computer-aided hyperparameter tuning approach using the ACO algorithm.

The overall picture of the hyperparameter selection algorithm using ACO method for CBCT reconstruction is shown in Figure 1. The detailed explanation of the method in this section is based on this diagram. Starting with the initialisation stage, the initial images are defined. These are previous iteration best image (prev-iter-best) and previous generation best image (prev-gen-best), which are both defined to be zero images as a starting point. The previous iteration best image (prev-iter-best) and the previous generation best image (prev-gen-best) are the reconstructed image with the highest score from the previous iteration and previous generation, respectively. Also, the pheromones of all values of hyperparameters are initially set to 1, such that the probability for an individual ant to choose any option is equal.

In the context of the problem in this work, the number of hyperparameters to be selected is five as required by the implementation of the AwPCSD algorithm. Apart from these, there are still two hyperparameters, data-inconsistency-tolerance parameter (*ε*) and TV sub-iteration number (ng), that require proper setting. The ACO algorithm is employed to identify the optimal hyperparameter setting for the AwPCSD reconstruction algorithm for a given set of limited projection data.

In each iteration of the algorithm, the process loops through each ant in the colony. The prev-iter-best is used as a base image for all ants. An individual ant chooses the value of each hyperparameter setting following the probability, which is computed based on the pheromone using the following equation:

(6) $$\mathrm{Pi}=\frac{\tau_{i}}{\sum_{q=0}^{R}\tau_{q}}$$

where *τ_i_* is the pheromone for the choice of hyperparameter value *i* and *R* is the total number of available choices for the hyperparameter. In the first iteration, the pheromones of all the hyperparameters are set to 1, making it possible for each ant in the first generation to freely choose any choices. Each ant then moves on to the reconstruction process, carrying choices of hyperparameters it has chosen. This process involves running several instances of the AwPCSD reconstruction algorithm. For each run of the AwPCSD algorithm, one iteration of the algorithm is performed. The reconstructed image from each ant is compared with the reference image and a correlation coefficient (CC) is computed and used as a score. In this study the reference image is a true image of the XCAT phantom model, in clinical studies where images with full projections are available, those could be used as a reference image instead. This step is repeated until all ants in a colony have finished their moves.

The reconstructed image with the highest score among all the ants in that generation is kept as the current generation best(current-gen-best) image result. After one generation of ants is finished, the score of each reconstructed image obtained from the reconstruction using each hyperparameter configuration is separately recorded for each hyperparameter value in that configuration. Then, all of the scores of each hyperparameter value in that generation of ants is averaged. This average score is used to update the pheromone for that choice of hyperparameter value for the next generation of the ant colony. The pheromone update equation is:

(7) $$\tau_{i}^{m+1}=\left(1-\sigma \right)\tau_{i}^{m}+{\bar{s}}_{i}$$

where ${\bar{s}}_{i}$ is the average score of all ants choosing the choice *i* of hyperparameters, *τ_i_^m^* is the pheromone of the choice *i* of hyperparameters for the *m*^th^ generation of ants and σ is the pheromone evaporation factor with a value between 0 and 1. The values of pheromones are normalised between 0 and 1. The pheromone for each choice of hyperparameter is updated based on the score obtained from the reconstructed image. This is to ensure that the choices which produce a highly-scored reconstructed image attract more ants in the next generation of ant colony. Then, two conditions are checked. The first condition checked is whether the score of the current-gen-best image is greater than that of the prev-gen-best image. If the answer is no, the algorithm launches another generation of ant colony replacing prev-gen-best with current-gen-best. If the answer is yes and the maximum number of iterations is not reached yet, the algorithm launches the new iteration and replacing prev-iter-best and prev-gen-best images with current-gen-best image. Apart from these two conditions, the stopping criteria as explained in Equations (8) and (9) are also checked for each reconstructed image. If these stopping criteria are met, the algorithm is stopped. Otherwise, the whole process is repeated until the user-defined maximum number of iterations is reached. At the end of the implementation, the hyperparameter configuration with the highest score is an optimal hyperparameter setting for a given data. Furthermore, the reconstructed image from this setting is obtained as an optimal result from the AwPCSD reconstruction algorithm.

The ACO approach in the proposed algorithm is able to identify optimal hyperparameters appropriate for a dataset and produce a good quality reconstructed image using limited numbers of projection data. The whole process does not require any knowledge about the iterative reconstruction algorithm from the user, nor the intervention during its implementation. The reconstructed image from the proposed algorithm is compared with the results from other ways of choosing parameters, visually and quantitatively. In addition, the optimal set of hyperparameters from the proposed algorithm is used to reconstruct images from the projection data with different levels of noise and different angle arrangements. The same set of experiments was also tested with different imaging samples to demonstrate the robustness of the proposed algorithm.

## 4. Results and Discussion

The experimental results and discussion are presented in this section. The details of the dataset used for the experiments are explained, as well as the quantifying metrics. Then, the proposed method was implemented in the training stage and the results were presented and compared with other algorithms. Finally, the set of hyperparameters were tested with different experimental settings.

### 4.1. The Digital 4D Extended Cardiac-Torso (XCAT) Phantom

**Figure 2:**
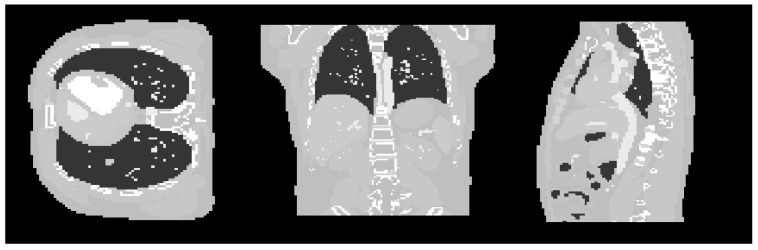
Cross-sectional slices of the XCAT phantom used for the training of parameter in the three axes.

In this work, datasets from the digital 4D Extended Cardiac-Torso (XCAT) Phantom [16,17] were used in the experiments. The thorax anatomy structure of the phantoms was selected to show the performance of the proposed algorithm. Three datasets simulated from the XCAT phantom were used for training and testing purposes. For the training, one dataset was used as shown in Figure 2. The chosen voxel size is 128 × 128 × 128.

Poisson and Gaussian noise [25,26] were added to the input projection data to simulate realistic noise. The case of default noise in the experiment was a combination of Poisson noise with maximum photon count of 60,000 and the Gaussian noise with mean and standard deviation of 0 and 0.5, respectively.

For the testing purpose, two different XCAT phantom datasets were generated with different parametrization from the training dataset. Further details are explained in sub-section of testing on different samples.

### 4.2. Evaluation Metrics

The evaluation metrics for an implementation of the proposed method, as well as a comparison with other algorithms are explained as follows:

#### 4.2.1. Correlation Coefficient (CC)

As mentioned in the methodology section, the CC metric is used to compute a score representing an imaging performance of particular hyperparameter configuration by comparing the reference image with the reconstructed image from each ant. The definition of *CC* metric can be expresses in the following equation:

(8) $$CC=\;\frac{Cov\left(\hat{f}\left(x\right),f\left(x\right)\right)}{\sigma_{\hat{f}\left(x\right)}\sigma_{f\left(x\right)}}$$

where *Cov*($\hat{f}$(*x*), *f*(*x*)) is the covariance between the true and reconstructed images, $\sigma_{\hat{f}\left(x\right)}$ is the standard deviation of the true image, *σ_f_*_(*x*__)_ is the standard deviation of the reconstructed image. The value of CC ranges from −1 to 1 where −1, 0 and 1 represent a total negative linear correlation, no linear correlation and total positive linear correlation, respectively.

#### 4.2.2. Universal Quality Index (UQI)

The UQI is one of the two metrics that was used to quantify the differences between image reconstruction results. This metric evaluates the degree of similarity between the reconstructed and reference images.The UQI can be described as:

(9) $$UQI=\frac{2cov\left(\mu ,\mu_{true}\right)}{\sigma^{2}+\sigma_{true}^{2}}\frac{2\bar{\mu }{\bar{\mu }}_{true}}{{\bar{\mu }}^{2}+{\bar{\mu }}_{true}^{2}}$$

where *µ* and *µ_true_* represent intensity of a reconstructed and a reference image, respectively, cov(·,·), *σ*^2^ and $\bar{\mu }$ are the covariance, variance and mean of intensities, respectively. A UQI value ranges from zero to one, where a value closer to one suggests better similarity to the reference image.

#### 4.2.3. Relative Error (*e*)

The relative error is another metric that is used to quantify the differences between the results. It can be computed as a relative 2-norm *error* using the equation below:

(10) $$relative\;error\;\left(e\right)=\frac{\|image-image_{ref}{\|}_{2}}{\|image_{ref}{\|}_{2}}$$

### 4.3. Training of Hyperparameters

For the training of hyperparameters using the proposed method, the following general parameters for the algorithm were defined: maximum number of iterations = 50, maximum number of generations of ant colony = 10, number of ants in a colony = 50, evaporation rate = 1. The configurations for the five hyperparameters in this experiment are displayed in Table 1.

The values of three hyperparameters are fixed, as explained earlier. These values remain constant for all hyperparameters configurations. There are 10 values for *ε* and 15 values for *ng.* The range of *ε* is chosen very wide to cover measurement and modelling error. Upper limit for sub iteration *ng* was chosen to allow for acceptable computational time when the parameter optimisation is completed. In total, there are 150 hyperparameter configurations to be optimised by the proposed algorithm. Each of an ant in a colony (in one generation) chooses the values of hyperparameters, *ε* and *ng*, to search for the optimal hyperparameter configuration for a given data. The performance of the proposed algorithm was evaluated in a limited data scenario by using the data set with 50 projection views, equally sampled from 0 to 360° with an increment of 7.2° between each angle. The testing computer used for the experiment was an Intel Core i7-4930K CPU at 3.40 GHz with 32 GB RAM. The single GPU in use is an NVDIA GeForce GT 610.

To evaluate the performance of the proposed method, the reconstructed image obtained from the optimal hyperparameters was compared with results from three other cases. The first case is the reconstruction result from the CGLS algorithm. The second case is the AwPCSD reconstruction algorithm using an arbitrary set of hyperparameters. The arbitrary set represents the way a user who is not familiar with the TV based CT reconstruction might have chosen this set of hyperparameters. The last case used for comparison is a cross-validation method [27], which is a technique that is used to evaluate predictive models by splitting the original data into training and testing sets. In one trial of cross-validation technique, a projection from one particular angle is removed from the available projection data. This projection is used as a testing data, while the remaining projections are used as a training set. The AwPCSD algorithm is implemented on the training set in parallel using each configuration of hyperparameters. Then, the reconstructed image obtained from each hyperparameter configuration is used to simulate the projection data of the same angle as that of the testing data that is taken out earlier. The root-mean-square error (RMSE) is measured between the simulated projection data and the testing data. Next, the cross-validation method moves on to the next trial, where the projection of adjacent angle is used as a testing data. The same procedure is repeated until the last angle of projection is used as a testing data. After all trials are finished, an average RMSE error of each hyperparameter configuration is computed. The best performing configuration is the one with the lowest average RMSE error and vice versa. The method is implemented in this work to evaluate the performance of the proposed method in terms of the quality of reconstruction, as well as the computational time.

Since the results of the cross-validation method are evaluated on testing data, which is not included in the data used to produce the results (training data), the cross-validation method is thus a good benchmark to compare with the proposed method. The proposed method, as well as the other three methods, were implemented with the simulated Extended Cardiac-Torso 4 (XCAT) phantom projection data, following the experimental setting explained in the next section.

**Figure 3:**
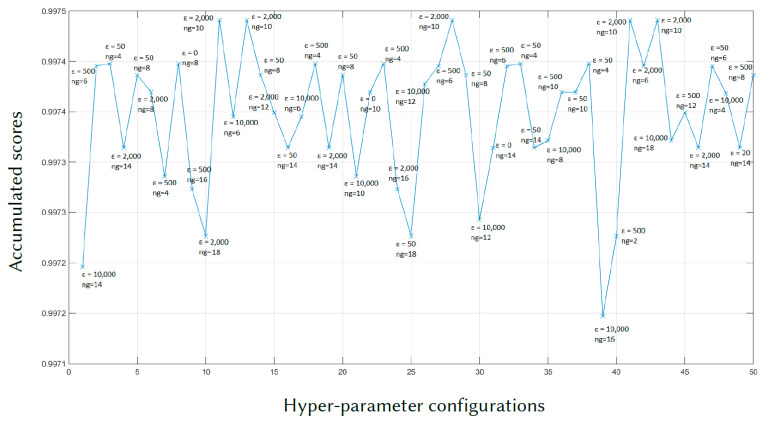
The plot of accumulated scores for all hyperparameter configurations at the end of the proposed method.

After all the iterations of the proposed algorithm, the scores of the hyperparameter configurations as chosen by ant colony in the last iteration are shown in Figure 3. These scores are computed based on accumulated probability over the entire implementation of the proposed method.

According to Figure 3, the hyperparameter configuration with the highest score is chosen as an optimal set of hyperparameters. The optimal set of hyperparameters found by the proposed and the cross-validation methods, as well as the arbitrary setting are displayed in Table 2. Note that for the CGLS algorithm, no hyperparameter is required. The maximum number of iteration for three cases is specified at 50. However, the CGLS algorithm converges and stops at the iteration number 15 and the AwPCSD algorithm with the arbitrary setting converges and stops at the iteration number 5.

**Figure 4:**
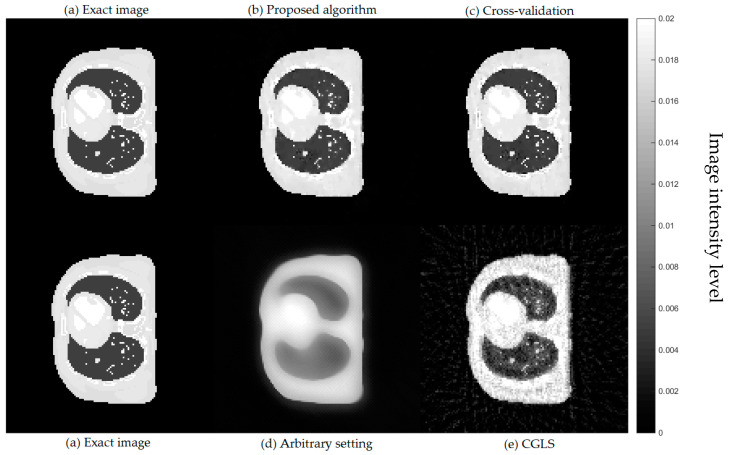
Cross-sectional slices of reconstructed images from 50 projection views obtained from four cases: (**a**) exact image; (**b**) the proposed algorithm; (**c**) cross-validation; (**d**) arbitrary setting; (**e**) CGLS. The display window is [0–0.02].

Comparing the computational time for all four cases, the CGLS algorithm is the one with the shortest time as it only takes a couple of seconds to finish its 15 iterations. For the AwPCSD algorithm with the arbitrary setting of hyperparameters, it takes approximately 2 min. The hyperparameter learning process using the proposed algorithm takes approximately 1.45 h to finish. The cross-validation algorithm is the one with the longest computational time. It takes approximately 47.15 h to finish the entire process. Cross-sectional slices of the reconstruction results from all cases are shown in Figure 4, in comparison with an exact phantom image.

**Figure 5:**
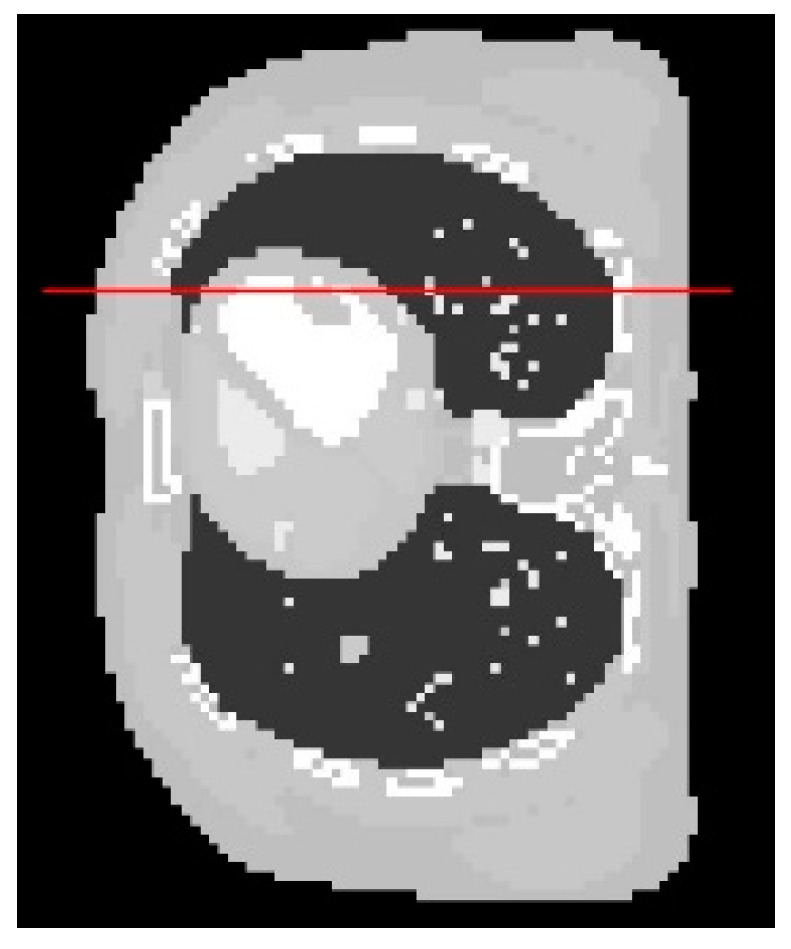
The image profiles of all the results along the horizontal line are plotted. The display window is [0–0.02].

**Figure 6:**
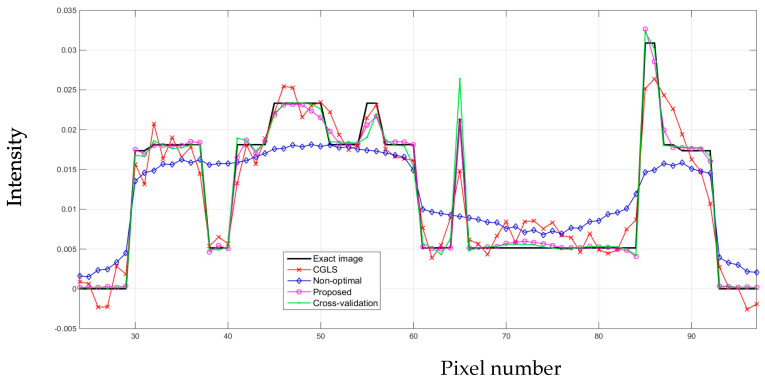
One-dimensional profiles of all the results from four cases, in comparison with the exact image.

From a visual inspection of Figure 4, the reconstructed image from the proposed algorithm and the cross-validation method are rather similar to each other. Not much outstanding difference can be observed in the images from both cases. However, they are ones that are the most similar to the exact phantom image, as they contain sharper edges than the blurry result from the arbitrary setting. Even though the CGLS algorithm is able to recover small features as well as edges, the image is relatively noisy compared to the result from the proposed algorithm and the cross-validation method. To make a comparison clearer, we analysed one-dimensional profile plots of all the results along an arbitrary row of a cross-sectional slice as shown in Figure 5. The 1D plot of all the results is shown in Figure 6, in comparison with the reference exact phantom.

According to the plots in Figure 6, the one-dimensional profiles from the proposed ACO algorithm and the cross-validation method are the most aligned with the profile from the exact image. This is particularly true around the edges, where the abrupt changes occur. In some areas, the profile from the proposed algorithm is more aligned to the exact image than that of the cross-validation method. But there are also some other areas where the cross-validation method is better aligned. Hence, the results from these two algorithms do not have any outstanding difference. In line with the visual inspection of Figure 4, the profile from the AwPCSD algorithm using the set of arbitrary hyperparameter settings shows that the algorithm failed to recover the edge information of the image. This can be seen between pixel numbers 30 to 60, where the profile plot is rather flat. The result from the CGLS is a middle ground between the first two cases. Although the CGLS algorithm is able to recover most of the image features, the result is much noisier compared to the proposed ACO algorithm. The result from the AwPCSD algorithm with the arbitrary setting can be improved further by re-selecting the values of hyperparameters and implement the algorithm again. It is a time-consuming and tedious process and there is no way to guarantee that the chosen hyperparameters will be the optimal ones. This highlights the significance and advantage of having the proposed computer-aided hyperparameter selection algorithm, which helps to save time and resource of the user to find an optimal set of hyperparameters.

### 4.4. Testing the Trained Hyperparameters

To evaluate the robustness of the proposed method, the optimal set of hyperparameters obtained from the training stage was used to reconstruct images in different scenarios. The details of each experiment are explained in the following sub-sections.

#### 4.4.1. Different Noise Levels

In the first test, the same dataset as that of the training stage was used, but three different noise levels were added to the projection dataset as explained in the following: *Noise 1 case*: Poisson noise = 30,000 maximum photon count and Gaussian noise with mean = 0, standard deviation of 1, *Noise 2 case*: Poisson noise = 20,000 maximum photon count and Gaussian noise with mean = 0, standard deviation of 3, *Noise 3 case*: Poisson noise = 10,000 maximum photon count and Gaussian noise with mean = 0, standard deviation of 5.

**Figure 7:**
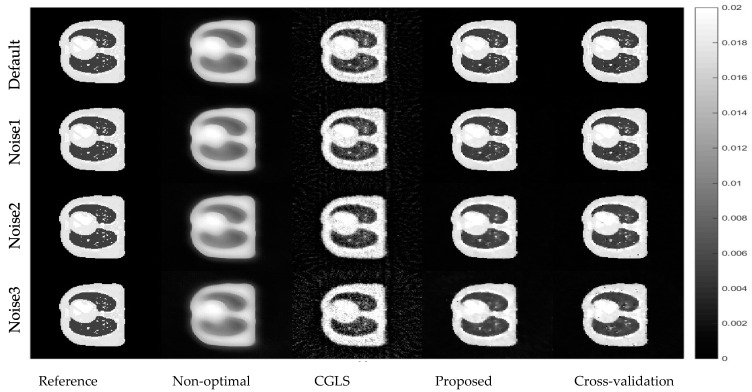
Reconstruction results from the projection data with different levels of noise. The display window is [0–0.02].

The AwPCSD algorithm was used to reconstruct the images by taking each of the noise level cases as an input. Cross-sectional slices of the reconstructed results in all cases are shown in Figure 7.

According to Figure 7 and Table 3, the results from the AwPCSD with arbitrary setting are clearly the worst among the others. This is because the cross-sectional slices are rather blurry, and the relative errors are the biggest with the lowest UQI values in all cases. The CGLS results are better than those of the arbitrary setting but are still corrupted by noise, getting worse as the noise level increases. Quantitatively, the relative errors and the UQI values show that the cross-validation method is in similar range to that of the proposed algorithm in all cases apart from relative error in the noise 3 case. New parameter tuning is able to produce images almost as good as those of the cross-validation method. As cross-validation method is much more computationally expensive in terms of the training time, this makes the method impractical in real use. The cross-validation method is implemented here for a comparison purpose. The point we are making in this experiment is to prove that the proposed algorithm is able to achieve almost the same quality of the result as that of the cross-validation method, but in a much more reasonable time frame. The experiment in this section proves that the same set of hyperparameters from the proposed method is robust against an increase of noise in the projection. The reconstructed images from the proposed algorithm are still able to maintain a superior quality over almost all other methods in all the noise cases.

#### 4.4.2. Different Sets of Projection Angles

**Figure 8:**
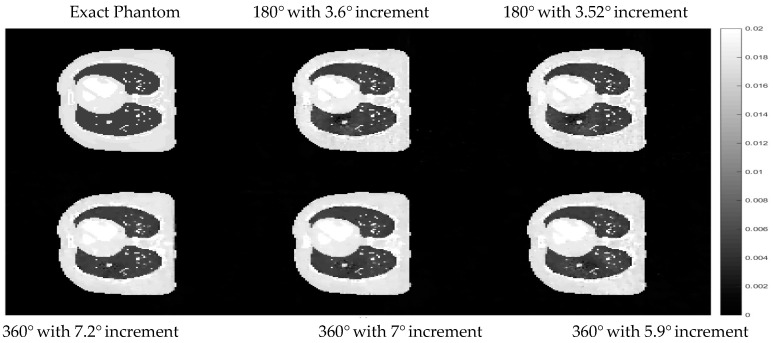
Cross-sectional slices of the reconstructed images with different angle arrangements. The display window is [0–0.02].

Further analysis with multiple arrangements of the angles used to acquire the projection data was implemented to ensure that the results are not unique to only one specific angle arrangement. In this test, the same dataset as that of the training stage was also used. The projection data used in the training of hyperparameters contains 50 projection views, equally sampled from 0 to 360° angle with an increment of 7.2° between each projection. This hyperparameter setting obtained from the training was tested with four other different angle arrangements with a fixed number of projections. The first two angle arrangements are the projection data collected over 360° with 7° and 5.9° increments, respectively. The other two arrangements are collected over 180°, with 3.6° and 3.52° increments. Cross-sectional slices of the reconstructed images with different angle arrangements using the same set of hyperparameters from the training stage of the proposed algorithm are shown in Figure 8.

**Figure 9:**
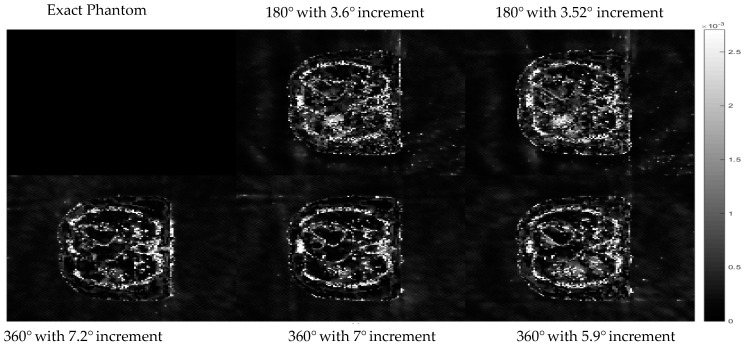
The difference between reconstructed images using proposed methods with reference data using the set of hyperparameters from the proposed algorithm with respect to different angle arrangement. The display window is [0–0.02].

The difference images between the cross-sectional slices in Figure 8 and the exact phantom are displayed in Figure 9 to better observe the differences in each case.

The results in Figure 8 and the difference images in Figure 9 show that the hyperparameter setting obtained from the proposed algorithm is able to reconstruct almost the same quality of image even when the angle arrangement of the projection data was changed.

#### 4.4.3. Different Samples

**Figure 10:**
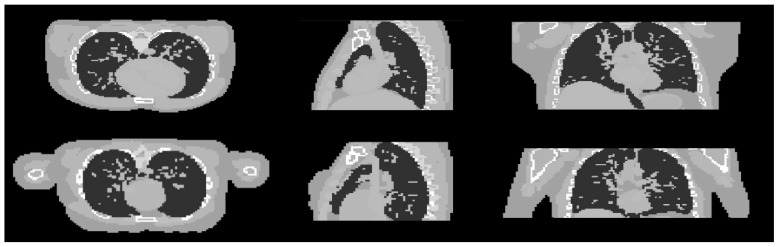
Cross sectional slices of the male (**top row**) and female (**bottom row**) phantoms in the three axes.

In this part, the robustness of the proposed algorithm was evaluated by applying the trained hyperparameter setting to the reconstruction of different samples. Two different XCAT phantom datasets were generated with different parametrisation from the training dataset. The first dataset is a male phantom with the chosen voxel size of 128 × 128 × 70. The second one is a female phantom with some modifications of the general parameters used to generate the phantom. The detail of the different modifications between these two phantoms is shown in Table 4. Cross-sectional slices of the two phantoms are shown in Figure 10.

In this experiment, the experimental setting used in the previous experiment with the first set of XCAT phantoms remains the same, i.e., the default case is 50 projection views, equally sampled from a 360° with Poisson noise of 60,000 maximum photon count and Gaussian noise with mean and standard deviation of 0 and 0.5, respectively.

**Figure 11:**
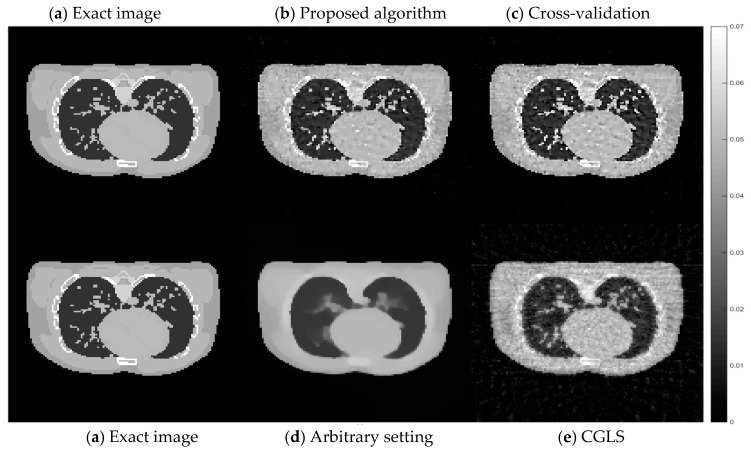
Cross-sectional slices of the reconstructed images from the male phantom using the same sets of hyperparameter settings and the CGLS algorithm as applied to the training dataset previously. The display window is [0–0.07].

In the same way, the same sets of hyperparameters used in the previous experiment as displayed in Table 2 were used to reconstruct images of the testing datasets, as well as the CGLS algorithm. The results of the male phantom are shown and discussed first. The cross-sectional images are shown in Figure 11.

**Figure 12:**
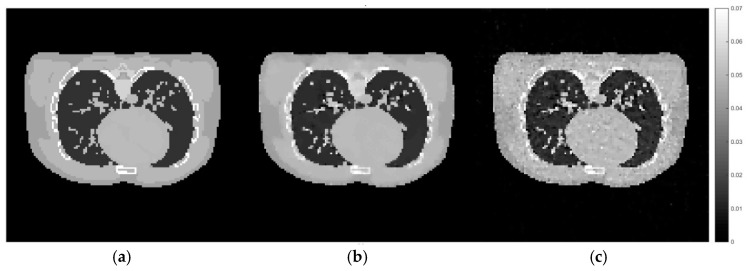
Cross-sectional slices of (**a**) the exact image of male phantom data, (**b**) the reconstruction from directly implemented the proposed method on the male phantom data, (**c**) the reconstruction using the hyperparameters obtained from the training stage.

According to Figure 11, the reconstruction using the set of hyperparameters obtained from the training dataset still able to produce good quality of image, comparing to other methods. We then implement the proposed method directly to the projection simulated from the male phantom to further analyse the difference between these two cases. The cross-sectional slices are shown in Figure 12. The relative errors and the UQI, as well as the sets of hyperparameters used in each method are presented in Table 5.

**Figure 13:**
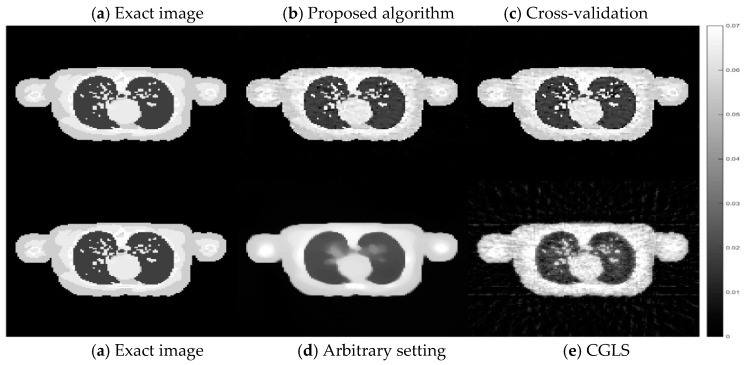
Cross-sectional slices of the reconstructed images from the female phantom using different method and hyperparameter settings. The display window is [0–0.07].

**Figure 14:**
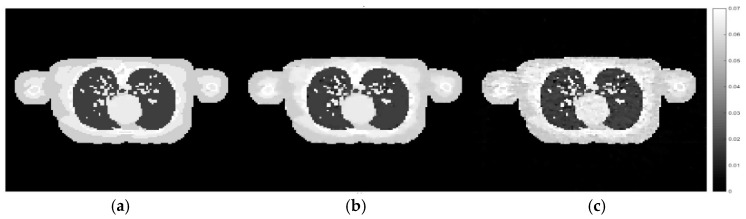
Cross-sectional slices of (**a**) the exact image, (**b**) the reconstruction from the proposed method directly implemented on the female phantom data, (**c**) the reconstruction using the set of hyperparameters from the training dataset. The display window is [0–0.07].

According to Figure 12 and Table 5, it can be concluded that the set of hyperparameters obtained from the proposed algorithm with the training set can be applied to different imaging samples and produce a result which is superior to CGLS and TV with arbitrary parameters. However, the set of hyperparameters obtained from directly implementing the proposed algorithm with the male phantom projection shows even better results. This experiment proves that the selection of hyperparameter for the TV regularisation algorithm, specifically for the AwPCSD algorithm, is data-specific. The optimal set of hyperparameters from one training dataset can still be applied to different image sampling within a similar context. However, the optimal result might not yet be achieved. It is significant to fine-tune the hyperparameters, in order to obtain the optimal result for a given data. This is the advantage that the proposed algorithm offers, to avoid the tedious process of manual hyperparameter tuning. The same pattern of experiment is performed on the female phantom and the results, as presented in the Figure 13 and Figure 14 and Table 6, confirm the conclusion stated above.

## 5. Conclusions

In this paper, a computer-aided hyperparameters optimisation algorithm for limited data CT reconstruction using the TV regularisation algorithm is proposed. In the proposed algorithm, the AwPCSD algorithm is used as a reconstruction algorithm. The ACO approach is employed to select the optimal set of hyperparameter for the reconstruction with the AwPCSD algorithm, which is crucial for the reconstruction result. Initially, the ranges of hyperparameter values are specified. The proposed algorithm searches through all possible configurations via a colony of ants and evaluates each configuration based on the score obtained from the comparison between the reconstructed image and the reference image. The pheromones are left for all configurations according to the scores, to attract ants in the next generation. At the end of the implementation, the set of hyperparameters with the highest score is considered as the optimal setting for a given projection data. The implementation of the proposed algorithm is fully automatic, without the need of human intervention during the processes. The experimental results showed that the images reconstructed using the proposed algorithm are superior to the results from CGLS algorithm and the AwPCSD algorithm using the arbitrary hyperparameter setting. Although the results of the proposed algorithm are slightly inferior to those of the cross-validation method as measured by the quantitative metrics, the computational time of the proposed algorithm is much shorter, being approximately over 10 times faster than the cross-validation method. Furthermore, the optimal set of hyperparameters from the training data is robust against an increase of noise in the projection data. The reconstructed images from the proposed algorithm are still able to maintain a superior quality over almost all the methods in all the noise cases. In addition, the optimal set of hyperparameters from one training dataset can still be applicable to different imaging samples with a similar context. Depending on the requirements of users in terms of imaging quality, the better result can be achieved by directly applying the proposed algorithm to the data. This is the advantage that the proposed algorithm offers, to avoid the tedious process of manual hyperparameter tuning. The computational time for ACO compared to a cross validation is also a significant advantage.

Finally, the limitations of this work should also be mentioned. Firstly, the implementation of TV-based regularization algorithms is highly dependent on hyperparameter values. The results presented in this work were based on some pre-defined values as mentioned in the experimental result section, i.e., *β* = 1, *β*_red_ = 0.99, *δ* = 0.0213. Although these values were taken from the conclusion of our previous work [7], different datasets may require different optimal values. The proposed method in this work was developed based on the configurations of two varying hyperparameters, in order to reduce the level of complexity for an initial proof of concept. Compared to our previous studies for parameter selection in [11], the proposed method in this work offers lower computational time while providing the same optimal parameters.

## 6. Future Work

The improvement of results for this work can be implemented in several directions. Firstly, the proposed method can be extended to include more/broader ranges of hyperparameters in its implementation. However, this comes at the cost of higher level of complexity and longer computational time. Secondly, an efficiency of the proposed method can be studied on a real CT measurement data, where the noise of the system is more realistic and unpredictable.

## Figures and Tables

**Table 1:** Hyperparameter configurations for this study.

Hyperparameters	Values
Data-inconsistency-tolerance parameter (*ε*)	0, 50, 70, 100, 200, 500, 2 × 10^3^, 1 × 10^4^, 1 × 10^5^, 5 × 10^5^
TV sub-iteration number (ng)	2, 4, 6, 8, 10, 12, 14, 16, 18, 20, 22, 24, 26, 28, 30
Relaxation parameter (*β*)	1
Reduction factor of relaxation parameter (*β*_red_)	0.99
Scale factor for adaptive-weighted TV norm (δ)	0.0213

**Table 2:** Different sets of hyperparameters used to compare the performance of the proposed algorithm.

Hyperparameter Selection Methods	*ε*	*ng*	*β*	*β*_red_	δ
Proposed algorithm	2000	10	1	0.99	0.0.0213
Arbitrary setting	700	100	1	0.99	0.0.0213
Cross-validation	0	8	1	0.99	0.0.0213

**Table 3:** Relative errors and UQI of image reconstruction results with XCAT Thorax phantom.

Case	e_cross_	e_proposed_	e_arbitrary_	e_CGLS_	UQI_cross_	UQI_proposed_	UQI_arbitrary_	UQI_CGLS_
Default	5.25%	6.70%	29.13%	19.26%	0.9970	0.9959	0.9322	0.9749
Noise 1	6.95%	8.97%	29.62%	20.64%	0.9959	0.9938	0.9293	0.9711
Noise 2	11.23%	13.32%	31.42%	23.22%	0.9905	0.9857	0.9182	0.9631
Noise 3	18.37	18.01%	32.86%	31.83%	0.9737	0.9717	0.9071	0.9330

**Table 4:** The parametrisation details of the two phantoms [11].

Parametrisation Details	Male	Female
motion option	beating heart only	respiratory only
length of beating heart cycle	1 s	5 s
starting phase of the heart	0.0	0.4
wall thickness for the left ventricle (LV)	non-uniform	uniform
LV end-systolic volume	0.0	0.5
start phase of the respiratory	0.0	0.4
anteroposterior diameter of the ribcage, body and lungs	0.5	1.2
heart’s lateral motion during breathing	0.0	0.5
heart’s up/down motion during breathing	2.0	3.0
breast type	prone	supine
factor to compress breast	half compression	no compression
thickness of sternum	~0.4 mm	0.6 mm
thickness of scapula	0.35 mm	0.55 mm
thickness of ribs	0.3 mm	0.5 mm
thickness of backbone	0.4 mm	0.6 mm

**Table 5:** Relative errors and UQI of image reconstruction results from the male phantom using each set of hyperparameters and the CGLS algorithm [15].

Sets of Hyperparameter/Method	Relative Error (%)	UQI	ε	*ng*
Proposed method from the training dataset	8.26	0.9946	2000	10
Proposed method with the male phantom	5.33	0.9980	70	22
Cross-validation from the training dataset	10.63	0.9909	0	8
Arbitrary setting	12.78	0.9863	700	100
CGLS	15.94	0.9798	N/A	N/A

**Table 6:** Relative errors and UQI of image reconstruction results from the female phantom using each set of hyperparameters and the CGLS algorithm [15].

Sets of Hyperparameter/Method	Relative Error (%)	UQI	ε	*ng*
Proposed method from the training dataset	7.52	0.9963	2000	10
Proposed method with the female phantom	5.55	0.9982	70	18
Cross-validation from the training dataset	8.84	0.9950	0	8
Arbitrary setting	16.70	0.9800	700	100
CGLS	19.35	0.9752	N/A	N/A
